# Proteomic Analysis Explores Interactions between *Lactiplantibacillus plantarum* and *Saccharomyces cerevisiae* during Sourdough Fermentation

**DOI:** 10.3390/microorganisms9112353

**Published:** 2021-11-14

**Authors:** Guohua Zhang, Qianhui Qi, Faizan Ahmed Sadiq, Wei Wang, Xiaxia He, Wei Wang

**Affiliations:** 1 School of Life Science, Shanxi University, Taiyuan 030006, China; 201923117002@email.sxu.edu.cn (Q.Q.); 202123117004@email.sxu.edu.cn (W.W.); 202123117002@email.sxu.edu.cn (X.H.); 2 State Key Laboratory of Food Science and Technology, Jiangnan University, Wuxi 214000, China; Faizan@jiangnan.edu.cn; 3 School of Food Science and Technology, Jiangnan University, Wuxi 214000, China; 4 Institute of Agr-Products Safety and Nutrition, Zhejiang Academy of Agricultural Sciences, Hangzhou 310021, China; 5 Key Laboratory of Information Traceability for Agricultural Products, Ministry of Agriculture and Rural Affairs of China, Hangzhou 310021, China

**Keywords:** sourdough, *Lactiplantibacillus plantarum*, *Saccharomyces cerevisiae*, proteomics, carbohydrate metabolism, amino acid metabolism

## Abstract

Sourdough is a fermentation culture which is formed following metabolic activities of a multiple bacterial and fungal species on raw dough. However, little is known about the mechanism of interaction among different species involved in fermentation. In this study, *Lactiplantibacillus plantarum* Sx3 and *Saccharomyces cerevisiae* Sq7 were selected. Protein changes in sourdough, fermented with single culture (either Sx3 or Sq7) and mixed culture (both Sx3 and Sq7), were evaluated by proteomics. The results show that carbohydrate metabolism in mixed-culture-based sourdough is the most important metabolic pathway. A greater abundance of L-lactate dehydrogenase and UDP-glucose 4-epimerase that contribute to the quality of sourdough were observed in mixed-culture-based sourdough than those produced by a single culture. Calreticulin, enolase, seryl-tRNA synthetase, ribosomal protein L23, ribosomal protein L16, and ribosomal protein L5 that are needed for the stability of proteins were increased in mixed-culture-based sourdough. The abundance of some compounds which play an important role in enhancing the nutritional characteristics and flavour of sourdough (citrate synthase, aldehyde dehydrogenase, pyruvate decarboxylase, pyruvate dehydrogenase E1 and acetyl-CoA) was decreased. In summary, this approach provided new insights into the interaction between *L. plantarum* and *S. cerevisiae* in sourdough, which may serve as a base for further research into the detailed mechanism.

## 1. Introduction

Sourdough is a traditional starter culture, which is used in making steamed buns, bread, and many other artisanal sourdough-based baked foods with unique flavours throughout the world [1,2]. The complex microbioflora of sourdough including yeasts, lactic acid bacteria (LAB), and moulds, have different metabolic capabilities and nutritional needs [3]. The distinctive flavour and puffy texture of sourdough is a result of lactic acid produced by LAB and CO_2_ produced by yeast [4,5]. Nowadays, approximately up to 50% of wheat is used to make steamed bread using sourdough in many Asian countries including China [6]. The use of sourdough is important for improving flavour, texture, shelf-life, and the high nutritional value of many cereal-based products incorporating sourdough as a functional ingredient [7].

Sourdough has been extensively studied worldwide for its unique flavour [8,9]. At present, more than 80 LAB and 50 yeast species have been isolated and identified. The dominant yeast species are *Saccharomyces cerevisiae*, *Candida humilis*, *Wickerhamomyces anomalus*, *Torulaspora delbruecki*, *Kazachstania humilis*, and *Pichia kudriavzevii*. However, *S. cerevisiae* is considered as the most crucial species of the sourdough ecosystem among all reported yeast species [10]. The main types of LAB include Lactiplantibacillus, Lactococcus, Leuconostoc, Weissella, Pediococcus, and Enterococcus species and among them, *Lactiplantibacillus plantarum* and *Lactobacillus sanfranciscensis* are the most dominant species [11]. Fermentable sugars are converted into ethanol and CO_2_ by yeasts in sourdough through the glycolytic pathway (EMP) and tricarboxylic acid cycle (TCA cycle), which make fermented products porous and puffy. During the EMP pathway, glucose is converted into pyruvate by some enzymes, such as hexokinase, phosphofructokinase, and pyruvate kinase. Then, pyruvate is converted into acetyl-CoA by pyruvate dehydrogenase complex, which is completely oxidized to CO_2_ in the TCA cycle [12].

Sourdough is characterized by low pH because of the abundant lactic acid and acetic acid produced during the fermentation, which it can enhance the flavours and ductility of the dough [13,14]. Extracellular polysaccharides, one of the important metabolites of LAB, can form a chemical bond between starch granules and the gluten network in wheat bran sourdough and establish a uniform network that positively ameliorates the adverse effects of acidification on the sourdough [15]. It is worth noting that the balance between the content of sugars and acids is the most important factor in the production of high-quality sourdough. Fermentation improves the volume, texture, flavour, and nutritional value of the dough. Controlled fermentation with carefully selected strains of LAB retards the staling process of bread and protects it from spoilage caused by moulds and bacteria—a problem that prevails in spontaneous fermentation [16]. Thus, it is necessary to study the role of *L. plantarum* in the fermentation process of sourdough. Many studies have proved that interactions between LAB and yeast are important to be elucidated in order to understand the characteristics of sourdough. Teleky B.E. et al. studied and compared the fermentation ecology and functional efficiency of LAB (*L. plantarum* ATCC 8014 and *L. casei ATCC 393*) with and without *S. cerevisiae* during sourdough fermentation, and observed that cocultures of LAB and yeast produced a higher quantity of lactic acid than single cultures [17]. The flavour of wheat bread could be enhanced by the concentration of free amino acids which depends on the composition of starter cultures in sourdough. An increase in the intensity of bread flavour was reported when preferments prepared with LAB were used to prepare sourdough [18]. Some mutualistic interactions between *L. plantarum* and *S. cerevisiae* have also been reported. When glucose, fructose and lactose were present as carbon sources, *L. plantarum* and *S. cerevisiae* stimulated each other. In addition, it was demonstrated that *L. sanfranciscensis* is stimulated by CO_2_ and another yet-to-be-identified factor produced by yeast in a sourdough-like environment [19]. Recent research has found that the ratio of LAB to yeast may affect the overall flavour of fermented products by influencing the Maillard reaction [13]. It has been verified that the spectrum and the relative abundance of volatiles was highest in the sourdoughs fermented by lactobacilli and yeast. Moreover, pyrazines, Maillard reaction products with “roasted” and “popcorn-like” flavour that are produced in the crust during baking, were associated with sourdoughs fermented by both yeasts and lactobacilli. The volatile profile of sourdough is rather complicated, and production of many other metabolites are dependent on interactions between LAB and yeasts. The profile of sourdough volatiles, especially esters, is reportedly more complicated when it fermented by a mixed culture comprising yeasts and LAB [20]. Nevertheless, interactions between *S. cerevisiae* and *L. plantarum* during the fermentation of sourdough and their consequences for the quality and aroma profile of a resultant sourdough have not been investigated yet—these factors are worth investigation.

In this study, single starter culture (either *L. plantarum* Sx3 or *S. cerevisiae* Sq7) and mixed starter culture including *L. plantarum* Sx3 and *S. cerevisiae* Sq7 were used for the sourdough making and microbial growth, viable cell counts, pH, and total titratable acidity (TTA) were determined. Finally, microbial community dynamics and key metabolic enzymes were analysed by high-throughput sequencing technology and proteomics technology. The results of this study can provide rationale to the selection of *S. cerevisiae* and *L. plantarum* as a sourdough starter culture for an improved fermentation process.

## 2. Materials and Methods

### 2.1. Fermentation and Growth Determination

*L. plantarum* Sx3 and *S. cerevisiae* Sq7 were isolated from a Chinese traditional sourdough sample and stored in the traditional fermented food laboratory, Shanxi University. Briefly, Sx3 and Sq7 were cultured overnight in maltose DeMan Rogosa Sharpe (mMRS) medium and Yeast Peptone Dextrose (YPD) medium at 30 °C under anaerobic and aerobic conditions, respectively [21,22]. Then, Sx3 and Sq7 were serially diluted to 10^−5^ and 100 μL bacterial solution was placed onto mMRS and YPD agar plates, and cultured for 48 h. Single colonies of Sx3 and Sq7 were picked and inoculated into 100 mL mMRS and YPD liquid media for 24 h, respectively. Then, 2% (*v*/*v*) precultured Sx3 and Sq7 were used to inoculate 450 g dough (300 g high-gluten wheat flour added to 150 mL water) as single-cultivated samples. Then, 2% precultured Sx3 and 2% precultured Sq7 were simultaneously inoculated into 450 g dough (300 g high-gluten wheat flour added to 150 g water) as cofermentation samples. All sourdough samples were incubated in an incubator at 30 °C.

Colonies were counted at the following fermentation time points: 0, 2, 4, 6, 8, 10, 12, 24, and 48 h. Single-cultivated Sx3 and Sq7 samples were plated onto mMRS and YPD agar plates, respectively. Meanwhile, cocultivated samples were plated onto mMRS agar plates with added cycloheximide (final concertation: 0.1 g/L) to inhibit the growth of Sq7 for counting Sx3 cells. In turn, cocultivated samples were plated onto YPD agar plates supplemented with chloromycetin (final concertation: 0.1 g/L) to inhibit the growth of Sx3 for counting Sq7 cells specifically. The dough without Sx3 and Sq7 was used as a control sample.

### 2.2. Determination of pH and TTA

10 g of each sourdough sample was added into 90 mL of distilled water followed by stirring for 10 min at medium speed until the sourdough sample was completely mixed. The pH value of the suspension was measured with a pH meter (FiveEasy Plus, Hangzhou, China). When the suspension was titrated with 0.1 mol/L NaOH solution to a pH value of 8.5, the volume of NaOH consumed (mL) was used to measure the TTA value of the sourdough sample [23]. 

### 2.3. Microbial Diversity

MO BIO’s PowerSoil DNA Isolation Kit (MO BIO-Laboratories, San Diego, CA, USA) was used to extract DNA from sourdough samples: control, single cultured samples, and cocultured samples. DNA purity and DNA concentration were detected by NanoDrop 2000 UV–vis spectrophotometer (Thermo Scientific, Wilmington, NC, USA). DNA integrity was detected using agarose gel electrophoresis. The samples were first melted on ice, mixed thoroughly, and centrifuged before an appropriate amount of the sample was taken for testing. The detection parameters are as follows: the PCR products of the same sample were mixed and detected by agarose gel electrophoresis using 1% agarose gel for 20 min with a running voltage of 5 V/cm. Primers were designed according to the designated sequencing region. The sequence regions of *L. plantarum* were amplified using the forward primer 338F (5′-ACTCCTACGGGAGGCAGCAG-3′) and the reverse primer 806R (5′-GGACTACHVGGGTWTCTAAT-3′). Whereas for yeast, the sequence regions were targeted using the forward primer ITS1F (5′-CTTGGTCATTTAGAGGAAGTAA-3′) and the reverse primer ITS2R (5′-GCTGCGTTCTTCATCGATGC-3′). To ensure the accuracy and reliability of subsequent data analysis, some samples were randomly selected to make sure that most of the samples in the lowest cycle number can amplify products with the right concentration.

After the preliminary experiment was completed, the PCR reaction system (TransGen AP221-02, 20 μL) consisting of 2.5 mM dNTPs, transstart fastpfu DNA polymerase, forward primer (5 μM), and reverse primer (5 μM), was run under the following conditions: a. 1× (3 min at 95 °C) b. cycle number× (30 s at 95 °C; 30 s at annealing temperature °C; 45 s at 72 °C) c. 10 min at 72 °C, 10 °C until halted by users. The PCR products were detected by 2% agarose gel electrophoresis detection. The PCR products were purified and three replicates per sample were prepared. The AxyPrepDNA gel recovery kit (AXYGEN) was used to cut the gel to recover the PCR products, and Tris-HCl was used to elute the staining solution. Then, electrophoresis was used for detection. Paired-end reads obtained by Illumina Miseq (Illumina, San Diego, CA, USA) sequencing were first spliced according to the overlapping relationship, while quality control and filtering were performed on the sequence quality [24]. Operational taxonomic unit cluster analysis and species taxonomy analysis were performed after processing samples. The bar graph of the community was obtained and the composition of the sourdough community was analysed based on the results of operational taxonomic unit cluster analysis. Genetic sequencing data have been deposited to the National Center of Biotechnology Information under SAR succession number SRP305571.

### 2.4. Protein Extraction

All dough samples including blank samples—the samples which were not fermented by any of the two strains, *S. cerevisiae*-based fermented sample or *L. plantarum*-based fermented sample, and the mixed sample stored in a freezer, were placed in shaker tubes. An appropriate amount of BPP solution (100 mM EDTA, 50 mM borax, 50 mM vitamin C, 30% sucrose, 100 mM TrisBase, 1% TritonX-100, 5 mM DTT, pH 8.0) was added. The high-throughput tissue mill was used to shake the samples 4 times for 40 s each time. The supernatant was taken followed by centrifugation at 12,000× g for 20 min at 4 °C and transferred to a 5 mL centrifuge tube. An equal volume of Tris-saturated phenol was added and the samples were vortexed at 4 °C for 10 min. The phenol phase was taken after centrifugation and mixed with BPP solution followed by vortexing at 4 °C for 10 min. The samples were centrifuged at 12,000× g for 20 min at 4 °C, and then the phenol phase was taken and mixed with a prechilled 0.1 M ammonium acetate methanol solution (5 times the volume of the phenol phase). The protein was precipitated overnight at −20 °C. On the following day, the obtained protein was centrifuged at 15,000× *g* for 20 min at 4 °C, and the supernatant was discarded. Then, 90% acetone was added into the precipitate and the solution was vortexed for 10 s, followed by centrifugation at 12,000× *g* for 20 min at 4 °C. The precipitate was then dissolved with a protein lysate (8 M urea + 1% SDS, containing the protease inhibitor), and centrifuged at 12,000× *g* for 20 min at 4 °C before taking the protein supernatant [25].

### 2.5. Protein Identification

Bicinchoninic acid (BCA) quantification [26] and SDS-PAGE electrophoresis were also performed. Reductive alkylation was performed on protein samples. Then, 100 μg of a protein sample was taken and triethylammonium bicarbonate buffer (TEAB) was added to make the final concentration of TEAB 100 mM. Then, Tris (2-carboxyethyl) phosphine (TCEP) was added to make the final concentration of TCEP 100 mM and allowed to react at 37 °C for 60 min. Iodoacetamide was added to make the final concentration of iodoacetamide 40 mM, and the reaction was allowed to continue for 40 min in the dark at room temperature, followed by the addition of precooled acetone (acetone: sample *v*/*v* = 6:1) to each tube, and precipitation at −20 °C for 4 h.

The samples were centrifuged at 10,000× *g* for 20 min. The precipitate was taken and dissolved with 100 µL of 100 mM TEAB thoroughly. Trypsin was added to the protein in a ratio of 1:50 by weight of protein, and the samples were hydrolysed overnight at 37 °C. Each sample was digested with the same amount of protein trypsin to quantify the peptides that had been digested. ThermoFisher Scientific (Cat. No.: 23275) peptide quantification kit was used for the peptide quantification. Based on the quantitative results, peptides were dissolved with an equal concentration of 0.25 μg/μL with mass spectrometry loading buffer (2% acetonitrile and 0.1% formic acid) for mass spectrometry (LC–MS/MS) analysis. 

KEGG orthology is a classification system that uses genes and their products to determine molecular functions. If the functions of orthologous genes and their products turn out to be similar and share a common metabolic pathway, they will be given the same KEGG orthology label. Therefore, this study used the KEGG Automatic Annotation Server (KAAS) software to classify target proteins into KEGG orthology. Then, the KEGG pathway information of the target protein participation was obtained by comparison with the database KEGG GENES. The mass-spectrometry proteomics data have been deposited to the ProteomeXchange Consortium via the IProX partner repository with the dataset identifier PXD024265. 

### 2.6. Statistical Analysis

Statistical analyses were performed by SPSS 17.0. Data were expressed as mean ± SD. The comparisons of means were determined by Tukey’s test (*p* ˂ 0.05). A total of three replicates were used for each sample and the experiment was repeated on three different occasions.

## 3. Results and Discussion

### 3.1. Viable Cell Counts, pH and TTA

**Figure 1:**
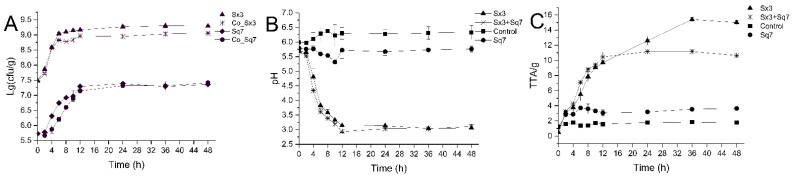
Cell counts of Sx3 and Sq7 in single culture and cocultivation (**A**), pH of sourdough (**B**), TTA of sourdough (**C**).

The viable cell counts of the two types of sourdoughs were compared (Figure 1A). As we can see, the growth trends of Sx3 and Sq7 were almost synchronous in the sourdoughs fermented by either single or mixed culture. At the beginning of fermentation (0 to 2 h), a slower growth rate of Sx3 and Sq7 was noticed in the cocultured sourdough compared with their growth in single-culture-based sourdough samples. Sx3 grew rapidly in cocultured sourdough from 2 to 4 h and achieved the same cell count (*p* > 0.05) as the sourdough sample fermented by Sx3 at 4 h of fermentation. This growth trend may be due to slow adaptability of bacterial species to each other in a new niche at the beginning. *L. plantarum* is one of the dominant LAB strains in the sourdough ecosystem [27], and this strain (Sx3) has well-adapted itself for growth in wheat-based sourdoughs. Sq7 maintained a slow growth state in the coculture fermentation, and the cell counts reached 10^7^ colony forming units (cfu) /g at 7 h of fermentation. For proteomics analysis, the sampling time was chosen at the exponential phase (fermented for 6 h). This ensured a high number of proteins in the metabolically active states of both strains. It can be seen from Figure 1 that the growth rate of Sq7 decreased during the coculture sourdough fermentation. 

The comparison of pH and TTA values among all sourdough samples is shown in Figure 1B,C. Certain phenomena were noticed in sourdoughs fermented by Sx3 culture alone and with the mixed culture containing Sx3—the pH dramatically decreased and the TTA increased during the first 12 h of fermentation. The pH of Sq7-fermented and control dough samples decreased slightly, and the TTA increased slightly. These changes coincided with the rapid growth of microbial cells, which can also be seen in several studies led on different substrates [17,28,29]. During EMP, the LAB convert glucose to pyruvate which is further dehydrogenated to lactic acid, and thus pH of the medium decreases and TTA increases via lactate dehydrogenase. After 12 h, TTA of the cocultured sourdough became stable gradually. However, there was still an increase in the TTA value of Sx3-fermented sourdough. This may be due to the production of some organic acids and other metabolites in the sourdough sample fermented by Sx3, while the Sq7 in the cocultured sourdough consumed the organic acid substances generated by Sx3, which increased the TTA of the single-culture sourdough while the pH was basically unchanged.

### 3.2. Microbial Diversity of Different Sourdough

**Figure 2:**
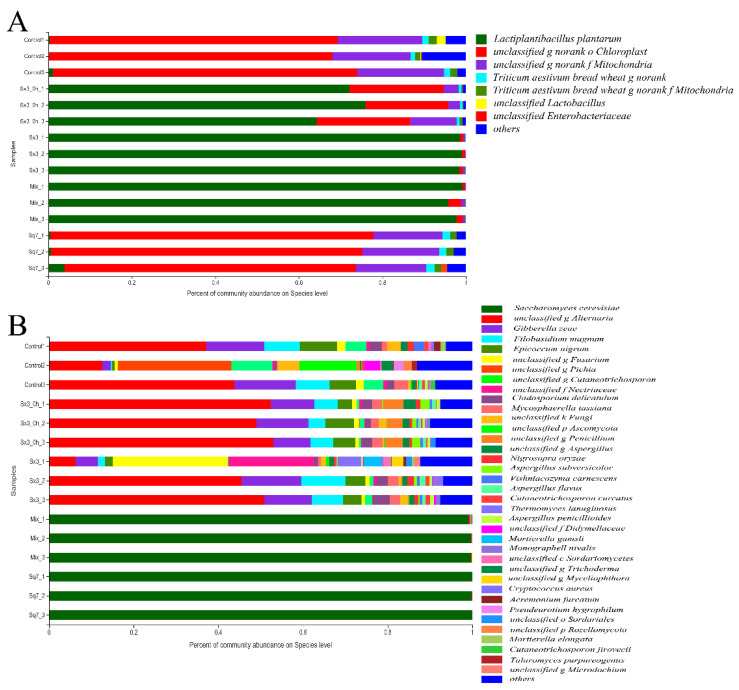
Diversity of bacteria (**A**) and fungi (**B**) in sourdough with 0 h fermentation (Sx3_0 h_1-3) and 6 h fermentation (Control_1-3, Sx3_1-3, Sq7_1-3, Mix_1-3).

High-throughput sequencing was used to analyse the distribution of bacteria and fungi at the species level. The microbial diversity identification results, as shown in Figure 2, revealed *L. plantarum* and *S. cerevisiae* as the most dominant organisms, which proved that the sourdough samples used in this experiment were not contaminated. In the bar chart of the bacterial community distribution (Figure 2A), *L. plantarum* was the absolute dominant bacterial species in the sourdough samples fermented by Sx3 culture alone and mixed culture at 0 h and 6 h of fermentation.

In the bar chart of the fungal community distribution (Figure 2B), the composition of fungal communities in sourdough was found to be more complex than that of bacteria. A total of 15 species with high abundance (relative abundance > 1% in at least one sample) were found in sourdoughs without Sq7 inoculation. In sourdoughs that had been fermented by only Sq7 culture and mixed culture for 6 h, *S. cerevisiae* was the only yeast species that prevailed in the dough samples. Both Sx3 and Sq7 showed growth advantage over other bacteria and fungi that were detected initially and then disappeared later. Thereby, their growth was not affected by the presence of other bacterial or fungal species. 

### 3.3. Proteomic

**Figure 3:**
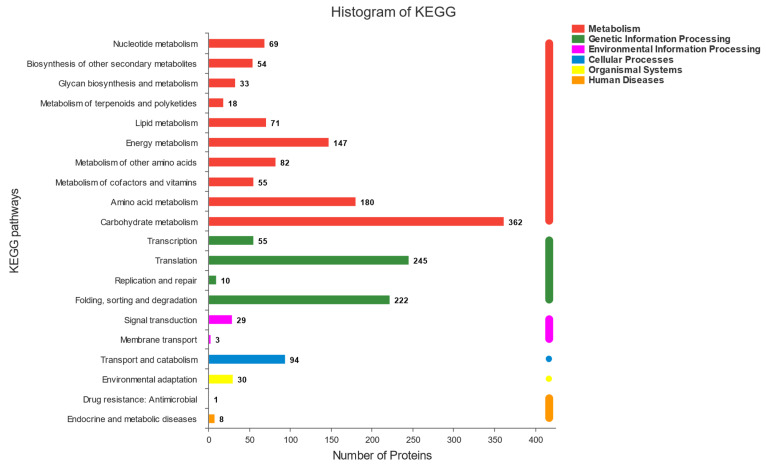
Whole protein function annotation.

The functional information of the proteins identified in this experiment was obtained comprehensively through six major databases (NR, Swiss-prot, Pfam, COG, GO, and KEGG databases). The annotations of each database were analysed statistically (Figure 3). There were three main metabolic pathways, related to the fermentation of sourdough with more than 100 proteins involved in each of them. Among these metabolic pathways, the highest number of proteins (362) were found to be involved in carbohydrate metabolism, followed by amino acid metabolism (180) and energy metabolism (147). The results revealed that the process of sourdough fermentation is almost equal tothe process of carbohydrate, amino acid, and energy metabolism. Interestingly, there were more than 400 proteins in the fermentation process related to protein translation, folding, sorting, and degradation. We believe that this is related to the formation of new proteins and the degradation of old proteins during sourdough fermentation. This experiment explored interactions between Sx3 and Sq7 based on carbohydrate metabolism, amino acid metabolism, protein formation, and degradation.

#### 3.3.1. Carbohydrate Metabolism

**Figure 4:**
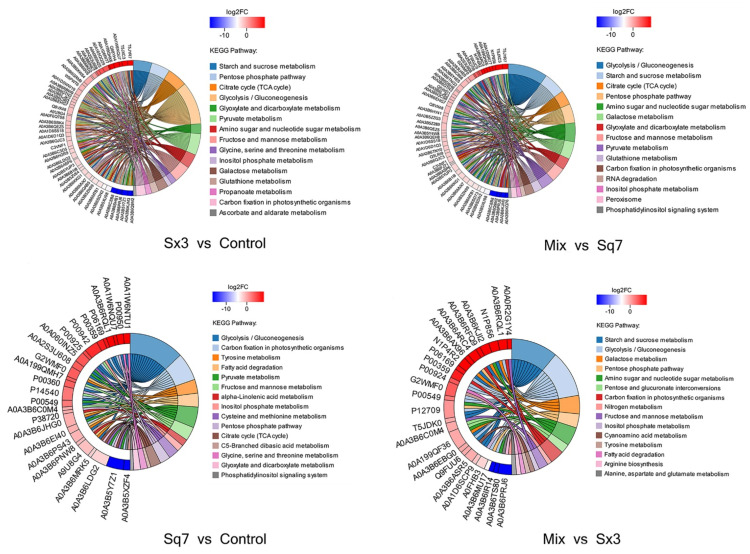
String diagram of protein enrichment in carbohydrate metabolism, the left side is the protein, showing log2FC in order from top to bottom. The larger the log2FC, the greater the fold difference in the expression of upregulated proteins. The smaller the log2FC, the larger the fold difference in the downregulated protein expression. The closer log2FC is to 0, the smaller the differential expression factor of the protein; the right is the name of the KEGG pathway that enriches the target protein.

In this study, KEGG functional enrichment analysis was performed on the proteins involved in carbohydrate metabolism, and the biological processes related to the proteins were determined at the functional level (Figure 4). In the process of carbohydrate metabolism, the following metabolic pathways were involved in four different fermentation types of sourdoughs: starch and sucrose metabolism, glycolysis, TCA cycle, pentose phosphate pathway, pyruvate metabolism, and amino sugar and nucleotide sugar metabolism. The fluctuation of protein content is based on the control sourdough. More proteins in sourdough fermented by Sx3 were significantly downregulated (86, *p* < 0.05) in each pathway of carbohydrate metabolism than were upregulated significantly (19, *p* < 0.05). In sourdough fermented by Sq7, the situation was opposite. There were more proteins (for instance, protein belonging to glycolysis, fructose and mannose metabolism) that were found significantly upregulated (16, *p* < 0.05) than downregulated (9). These results indicated that Sx3 plays a more important role in the fermentation of sourdough.

##### Starch and Sucrose Metabolism

In the process of starch and sucrose metabolism, sucrose synthase was downregulated in the Sx3-fermented sourdough sample, which shows the inhibition of the production of sucrose in the corresponding sample. However, sucrose synthase was upregulated in the mixed-culture sourdough, which shows the production of sucrose in the sourdough sample fermented by both species together. Sucrose increases the sweetness of sourdough as a natural edible sweetener, and also serves as a nutrient for the yeast to provide energy for the fermentation process [30]. Therefore, the upregulated sucrose synthase might have a positive impact on the mixed-culture sourdough. It is believed that sucrose delays the gelatinization of starch granules by absorbing water required by starch for gelatinization—a property beneficial for the preparation of bread and noodles [31]. Amylose and trehalose contents were increased in the sourdough fermented by mixed culture compared to those of single culture (either Sx3 or Sq7). The particle size and gelatinization characteristics of amylose have a great influence on the quality characteristics of sourdough, such as taste, whiteness, volume, and cooking quality. An increase in enzymes that regulate amylose might lead to a decrease in the expansion rate of sourdough and improve the elasticity, which means a reduction in losses associated with cooking and higher chewability of sourdough products [32]. Trehalose can prevent starch aging, protein denaturation, and lipid oxidative deterioration. In addition, it is crucial for the sweetness and aroma of sourdough [33]. Trehalose has been reported to protect *S. cerevisiae* cells against environmental stresses [34]

In the sourdough sample fermented by Sx3 alone, the downregulation of UDP-glucose 4-epimerase (galE) leads to a decrease in UDP-galactose content. The galE catalyses NAD+-dependent oxidoreductive interconversion of gluco- and galacto-hexoses (C4-epimerization) linked to UDP [35]. The galE plays a critical role in dietary galactose metabolism, endogenous galactose production and glycoprotein, and glycolipid biosynthesis [36]. In mixed-culture sourdough, galE was upregulated, which may be an effect caused by Sq7. Galactose is usually used as a nutritional sweetener because it contains high calories. Previous research has demonstrated that *L. plantarum* can effectively metabolize galactose and thus no galactose is produced in the sourdough fermented by only *L. plantarum* [37]. Therefore, cocultivation of yeast Sq7 and LAB strain (Sx3) can increase the sweetness of sourdough and improve the taste. At the same time, Sx3 can metabolize some galactose produced as a result of cocultivation by the Sq7 and use it for other metabolic activities.

##### Glycolysis

**Figure 5:**
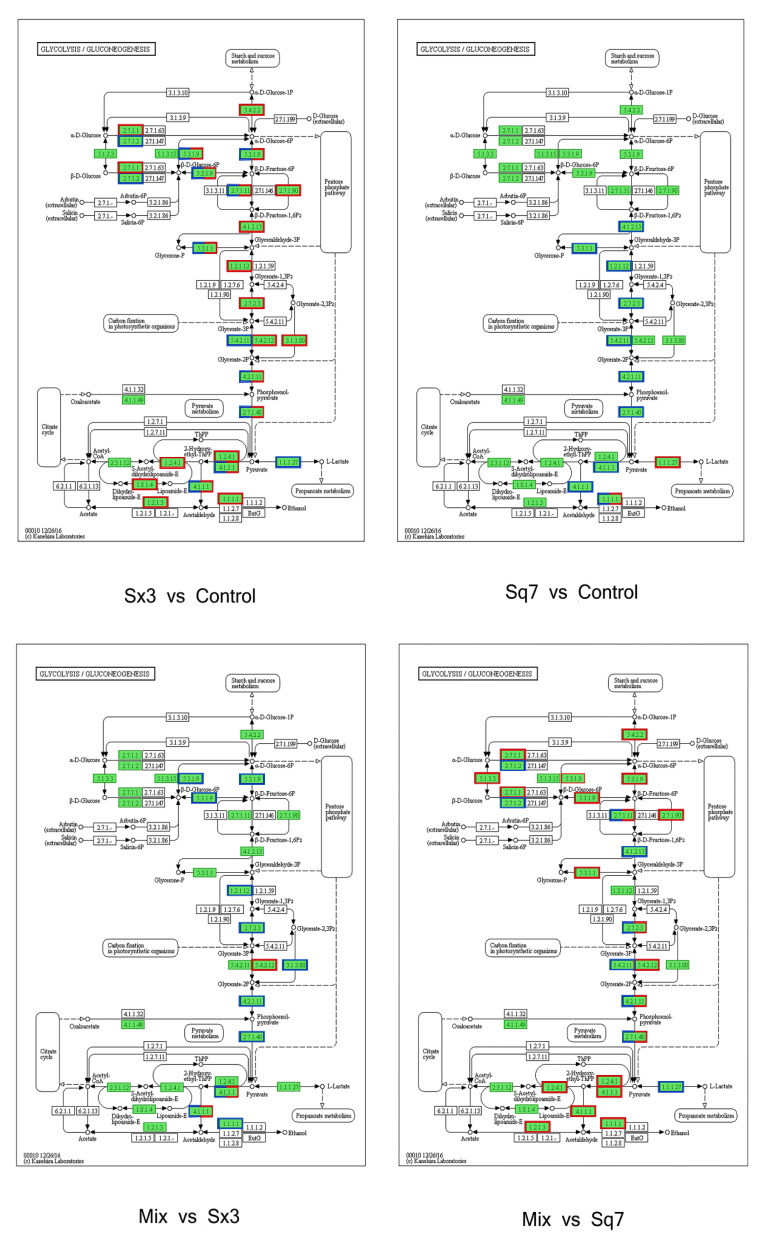
Glycolysis KEGG function annotation, the enzymes coloured in blue represent upregulated, in red represent downregulated enzymes according to proteomic data.

Low-molecular-weight sugars such as glucose and fructose are converted to pyruvate, and ATP is produced as an energy source during glycolysis (Figure 5). The changes in enzymes may affect the formation of downstream metabolites involved in many metabolic pathways. In the Sq7 single-culture sourdough, upregulation of fructose-bisphosphate aldolase activated the process of the conversion of β-d-fructose-1, 6P2 to glyceraldehyde-3P, which led to the upregulation of the relevant enzymes that generate pyruvate. At the same time, the upregulation of triose-phosphate isomerase may promote the formation of glycerone-P. The downregulation of L-lactate dehydrogenase (LDH) inhibited the production of L-lactate. The upregulation of pyruvate decarboxylase (PDC) promoted the production of acetaldehyde. In comparison to Sq7 single-culture sourdough, sourdough fermented by mixed culture showed the inhibition of the glycolysis pathway, leading to decreased pyruvate content, which was probably due to the presence of Sx3. However, aldehyde dehydrogenase (ALD) and upstream 6-phosphofructokinase-1 (PFK-1) were still upregulated. The TCA cycle in the mixed-culture sourdough group was inhibited, leading to a decrease in ATP concentration, as ATP is an allosteric inhibitor of PFK-1, whereas an increase in the concentration of AMP and ADP led to allosteric activation of PFK-1, leading to accelerated sugar decomposition [38]. The increase in LDH promoted the formation of L-lactate, which plays an important role in improving the flavour of sourdough, extending the shelf life, and enhancing the acidity. The downregulation of PDC in mixed-culture sourdough inhibited the formation of acetaldehyde, thereby inhibiting the activities of alcohol dehydrogenase (AD) and ALD, which may cause a decrease in ethanol and acetate contents. Reduction in acetate concentration helped to regulate the acidity during the fermentation process of sourdough. At the same time, excessive acetate may have a toxicological effect [39]. Mixed fungal and bacterial fermentation reduces acetate content and thus ensures the taste and quality of sourdough. In addition, the above-mentioned changes in enzymes as a result of mixed-species fermentation suppressed ethanol production. which means less ethanol stress during the fermentation of sourdough which is beneficial for the survival of Sx3. Partial inhibition of the glycolysis process during mixed fermentation may lead to less CO_2_ production, which means that there will be no breakage of pores and shrinkage in volume due to excessive gas production during fermentation [40].

Compared with the Sx3 single-culture sourdough, the enzyme promoting ethanol formation in was increased mixed-culture sourdough. Ethanol is an important flavour component in the fermentation process of sourdough. Ethanol can also react with the acetic acid produced by Sx3 to produce more ethyl acetate. At the same time, enzymes devoted to the production of acetaldehyde were also upregulated. Aldehydes and esters are also important flavour substances in sourdough, thereby increasing the richness of sourdough flavour. In mixed-culture sourdough, AD was inhibited compared with the sourdough fermented by Sq7, which shows that sourdough fermentation with the strains used in this study can help to improve the fermented product’s flavour, and also, it will not cause ethanol stress effect due to the excessive ethanol content. Upregulation of PDC in mixed-culture sourdough promoted the formation of acetaldehyde, which has a role in increasing the activity of ALD, thereby increasing the content of acetate. Acetate was considered to be an important enhancer of flavour compounds. It can also be used as a catalyst for the Maillard reaction with L-lactate [41]. The upregulated sucrose synthase in mixed-culture sourdough might have promoted the growth of Sx3, leading to enhanced production of L-lactate and acetate [42], thus it can be seen that mixed-culture fermentation, comprising the two species used in this study, can better control acetate content in sourdough compared to single-culture fermentation. Acetate content was regulated in the mixed-cultured sourdough sample so that it was enough for glycolysis.

In conclusion, LDH, PDC, AD, ALD, PFK-1 are the key enzymes in the glycolysis of carbohydrate metabolism. Pyruvate, L-lactate, ethanol and acetate, which play an important role in sourdough flavour, are regulated by these enzymes. The mixed cultured sourdough is better at controlling the flavour of sourdough in comparison with a sourdough fermented by either of the two strains used in this study alone. 

##### TCA-Cycle of Mix Sourdough

**Figure 6:**
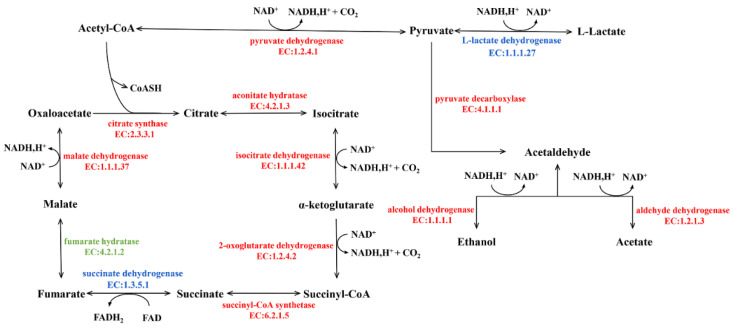
TCA-cycle of Mix vs Sq7, the enzymes coloured in blue represent upregulated, in red represent downregulated enzymes, in green represent the enzyme is no significant difference.

In comparing the Sq7 single-culture sourdough with the control, no difference in the TCA cycle was observed. In sourdough fermented with the mixed starter culture, there was one upregulated and six downregulated proteins or enzymes related to the TCA-cycle pathway (Figure 6). The LDH decreased in cocultivated sourdough during glycolysis and led to the inhibition of pyruvate dehydrogenase E1 component (AceE) activity, resulting in a decrease in acetyl-CoA content. Citrate synthase (CS) is a rate-limiting enzyme in the TCA cycle. The inhibition of CS activity slowed down the catalysed condensation reaction of oxaloacetate and acetyl-CoA in mixed-culture sourdough, which negatively impacts the entire TCA cycle, thereby inhibiting the production of succinic acid and malic acid downstream of citric acid. The reduction of organic acids such as citric acid or malic acid in the fermentation of mixed sourdough dough can create the best acidic environment. On the one hand, it can increase the antibacterial activity in sourdough, on the other hand, it can improve the activity of amylase and other enzymes and delay the retrogradation of starch. At the same time, a suitable acidic environment can hydrolyse protein and accumulate amino acids; these amino acids could undergo the Maillard reaction during the cooking process, and it can have higher elasticity, ductility and specific volume, and increase the sensory quality of sourdough [43]. A previous study has indicated a positive correlation between the glucose dehydrogenase (GDH) activity in lactic acid bacteria and their ability to metabolize amino acids. Co-metabolism of citric acid and glutamate can result in higher amino acid conversion rates [44]. Finally, organic acids can improve the oxidation resistance of sourdough. The appropriate amount of citric acid in sourdough improves the taste of sourdough and promotes appetite, but its high quantity can promote the excretion and deposition of calcium in the body. Eating foods containing a high quantity of citric acid for a long time may cause hypocalcaemia and increase the probability of duodenal cancer [4]. We suppose that making sourdough starter culture using the two strains used in this study can regulate citric acid content in sourdough, which may cause harm to the human body. Upregulation of succinate dehydrogenase (SD) confirms the formation of fumaric acid, which increases the acidity of sourdough and serves as a bacteriostatic and fungistatic agent. In addition, it is an important flavour substances in sourdough [43]. 

#### 3.3.2. Amino Acid Metabolism

**Figure 7:**
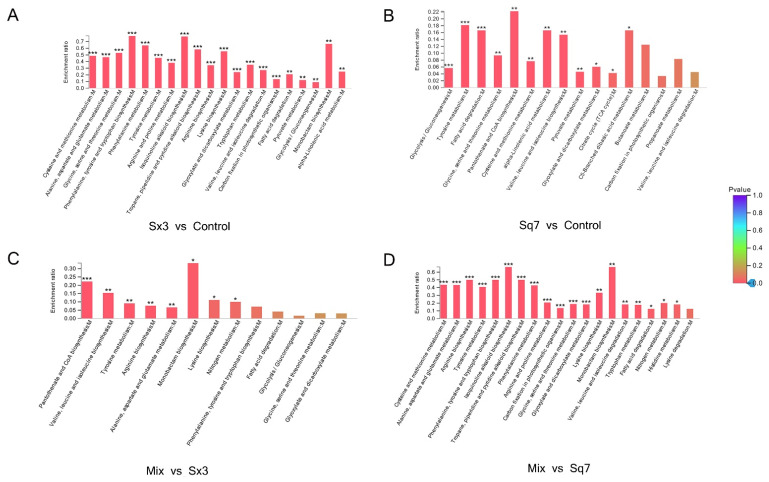
KEGG enrichment diagram of amino acid metabolism. The abscissa represents pathway name, and the ordinate represents enrichment rate (refers to the ratio of the protein number enriched in this pathway to the background number of the protein annotated into this pathway; the higher the ratio, the greater the degree of enrichment). The color gradient of the column represents the significance of enrichment. The deeper the default color is, the more significant the enrichment of the KEGG term is. “***” represents *p* < 0.001, “**” represents *p* < 0.01, and “*” represents *p* < 0.05.

The flavour of sourdough depends on the concentration of free amino acids [18]. LAB require many free amino acids for their growth and metabolism [44]. However, free amino acids are not abundant for the growth of LAB at the early stage of sourdough fermentation, thus the bacteria need an exogenous support which is probably the proteolysis of gliadin and glutenin, the most abundant proteins in sourdough and the main source of amino acids [45]. For amino acid metabolism (Figure 7), Sq7 single-culture sourdough was not significantly (*p* > 0.05) different from the control sourdough sample. However, in the Sx3 single-culture sourdough, there were 6 significantly (*p* < 0.05) upregulated proteins and 57 downregulated proteins. Since the regulation of amino acid metabolism in mixed-culture sourdough was mainly affected by Sx3, it was speculated that the difference in proteins between mixed-culture sourdough and Sx3 single-culture sourdough was not significant (*p* > 0.05), and the difference in proteins between mixed-culture sourdough and Sq7 single-culture sourdough was significant (*p* < 0.05), which was consistent with the sequencing results.

Amino acid metabolism was mainly related to alanine, aspartate, glutamate, glycine, serine, threonine, cysteine, methionine, and tyrosine. In comparison with the control sample, downregulated proteins or enzymes in Sx3 single-culture sourdough may lead to a decrease in the contents of amino acids, such as tyrosine, serine, glycine, alanine, and aspartate. The downregulation of glutamine synthetase inhibits the production of l-glutamate, whereas, upregulation of glutamine-fructose-6-phosphate transaminase promotes the conversion of l-glutamate to d-glucosamine-6P, which further promotes the formation of d-Glucosamine-6P, which indirectly affects the metabolism of the amino sugars. Vermeulen et al. [46] reported that in the combination of *L. plantarum* and *L. sanfranciscensis*, the enzyme reaction of NADH is strongly affected by citric acid and fructose. These cosubstrates can promote cofactor regeneration, so it is possible to increase the conversion rate of amino acids by increasing GDH activity. Affected by Sx3, the enzyme or protein changes in the metabolic process of mixed-culture sourdough compared with Sq7 single-culture sourdough were the same as Sx3-based sourdough. The downregulation of glycine hydroxymethyl transferase (glyA) and alanine-glyoxylate transaminase (AGXT2), in Sx3 single-culture sourdough, resulted in inhibition in serine and glycine in comparison to mixed-culture sourdough. There was no significant change in glyA and AGXT2 in mixed-culture sourdough, probably because of the effect of Sq7 in mixed-culture sourdough, which reflects the important role of yeast in regulating the content of serine and glycine. The anabolism of serine and glycine directly affects the production of glycerate-3-phosphate and pyruvate during glycolysis. Serine can be converted to pyruvate by serine deaminase [47], and a decrease in serine content may affect pyruvate biosynthesis, which is associated with reduced pyruvate production during pyruvate metabolism. Significant changes in amino acid metabolism show that *L. plantarum* affects many amino acid-related metabolic pathways in adapting fermented food environments.

#### 3.3.3. Protein Translation

Enzymes or proteins related to ribosome and aminoacyl-tRNA biosynthesis were actively expressed. Seryl-tRNA synthetase was downregulated in Sq7-based single-culture sourdough compared with control sourdough, while the seryl-tRNA synthetase was upregulated in mixed-culture sourdough, which might be due to the effect of Sx3. The tRNA synthetases are responsible for decoding the molecular information, from codons to amino acids. Seryl-tRNA synthetase aminoacylates the tRNA ^[*Ser*]Sec^ by two consecutive reactions. The first results in the formation of the l-seryladenylate intermediate releasing inorganic pyrophosphate (PPi) in the presence of ATP and Mg^2+^. The L-seryl group is then transferred to tRNA^Ser^, or tRNA^[*Ser*]Sec^, at the 3′-OH end, resulting in L-seryl-tRNA^Ser^ or L-seryl-tRNA^[*Ser*]Sec^ [48]. Mocibob et al. [49] found that the proximal region of seryl-tRNA synthetase C-terminal extension region can keep the protein which is generally stable. It was speculated that seryl-tRNA synthetase improved the stability of proteins during fermentation.

In Sx3 single-culture sourdough, a total of 28 and 18 subunit proteins were up- and down-regulated (Table 1), respectively, which indicated that DNA repair, cell development regulation, cell differentiation, and protein biosynthesis were more active during the fermentation of sourdough. Similarly, there were 19 upregulated subunit proteins and 2 downregulated subunit proteins in Sq7 single-culture sourdough. There were no significant changes (*p* > 0.05) in ribosomal subunit protein in mixed-culture sourdough compared with Sq7 single-culture sourdough. Among the ribosomal subunit proteins, RPL16, RPL5, and RPL23 did not change significantly (*p* > 0.05) during the fermentation of both Sx3 and Sq7 single-culture sourdough. During the fermentation of mixed-culture sourdough, the aforementioned proteins were upregulated, whereas RPS12 was downregulated. Majid et al. [50] reported high hydrophilicity of RPL16, in addition to having a high acid strength and thermal stability. Therefore, it was speculated that the upregulation of RPL16 may affect some metabolic pathways in the fermentation process of sourdough, which needs further research. The RPL23 gene is an essential gene in cell function, which plays an important role in maintaining protein stability, thereby promoting protein transport [51].

#### 3.3.4. Protein Folding, Sorting and Degradation

Calreticulin (or calregulin) is one of the major calcium-binding proteins of the endoplasmic reticulum (ER). It acts as a calcium-binding partner in the ER and participates in various biological processes. In Sq7 single-culture sourdough, calreticulin was inhibited, while it was upregulated in mixed-culture sourdough, where it might have helped coordinate the correct folding and transportation of some membrane surface proteins (exocrine proteins or endoplasmic reticulum-resident proteins) and in maintaining intracellular Ca^2+^ homeostasis [52]. Enolase is a key enzyme in glycolysis, which can catalyse the conversion of 3-phosphoglycerate to phosphoenolpyruvate (PEP). The upregulation of enolase in mixed-culture sourdough led to an increase in PEP content. PEP is catalysed to generate ATP and pyruvate by pyruvate kinase, thereby releasing energy.

## 4. Conclusions

This is the first study that has explored the interactions between *S. cerevisiae* Sq7 and *L. plantarum* Sx3 during sourdough fermentation through proteomics. This study has shown that carbohydrate metabolism is the most important metabolic pathway during the fermentation of mixed-culture sourdough when compared with single-cultured sourdough. Upregulated LDH might enhance the flavour of the sourdough, extend the shelf life and increase the sourness and refreshing taste. Upregulated SD might enhance the acidity of the sourdough. Downregulated CS in the mixed-culture sourdough might lead to a decrease in citric acid. Sucrose synthase was upregulated which may enhance the sweetness of sourdough. In mixed-culture sourdough, seryl-tRNA syn-thetase was upregulated, which probably improves the stability of proteins during fermentation. Sx3 and Sq7 cocultivation led to a decrease in the contents of tyrosine, serine, glycine, alanine, and aspartic acid. Ribosomal subunit protein plays an important role in maintaining protein structure stability in sourdough and promoting protein transport. In summary, the interaction between *L. plantarum* and *S. cerevisiae* has an important impact on the process of sourdough fermentation. This study lays a foundation for further research on the interaction between *L. plantarum* and *S. cerevisiae* and other microbiota of sourdough, for understanding more detailed mechanisms. In addition, it provides a rationale for the selection of *S. cerevisiae* and *L. plantarum* as a sourdough starter culture for an improved fermentation process. These results lay a foundation for the food industry to develop new starter culture rather than only relying on yeast in sourdough, to improve the flavour of bread and steamed buns.

## Figures and Tables

**Table 1:** Protein expression during the translation of different fermented sourdough samples.

Accession	Protein	Sx3 vs. Control	Sq7 vs. Control	Mix vs. Sx3	Mix vs. Sq7
Q88XY4	Ribosomal Protein LW	up	-	-	-
Q88XY3	Ribosomal Protein L2	up	-	-	-
Q88XY2	Ribosomal Protein S19	up	up	-	-
A0A5R1Q4J0	Ribosomal Protein L22	up	-	-	-
Q88XY9	Ribosomal Protein L16	-	-	up	-
P38701	Ribosomal Protein S20	up	up	-	-
Q01855	Ribosomal Protein S15	-	up	-	-
Q88XX6	Ribosomal Protein L14	up	up	-	-
Q88XY4	Ribosomal Protein L5	-	-	up	-
A0A151G4G6	Ribosomal Protein S8	up	-	-	-
A0A199QKN2	Ribosomal Protein L6	up	-	-	-
Q88XW8	Ribosomal Protein L30	up	-	-	-
W5BFB7	Ribosomal Protein S11	up	-	-	-
W5E6W1	Ribosomal Protein L23	-	-	up	up
P41057	Ribosomal Protein S29	-	up	-	-
W5FEV1	Ribosomal Protein L7	up	-	-	-
T5JQX3	Ribosomal Protein S13	up	-	-	-
Q88XW1	Ribosomal Protein SK	up	-	-	-
A0A199QHM8	Ribosomal Protein S4	up	-	-	-
A0A5R9DK27	Ribosomal Protein L17	up	-	-	-
A0A1W6NPG2	Ribosomal Protein L13	up	-	up	-
Q88XU7	Ribosomal Protein S9	up	up	-	-
P39516	Ribosomal Protein S14	-	up	-	-
B3LM31	Ribosomal Protein S16	-	up	-	-
Q88XY9	Ribosomal Protein S7	up	-	-	-
Q88YW7	Ribosomal Protein L7	up	up	-	-
Q88YW8	Ribosomal Protein L10	up	-	-	-
T5JY27	Ribosomal Protein L11	up	up	up	-
P0CX53	Ribosomal Protein L12	-	up	-	-
A0A484HZQ2	Ribosomal Protein S2	up	-	-	-
A0A165WB37	Ribosomal Protein L31	up	-	-	-
A0A2S3U801	Ribosomal Protein L33	up	-	-	-
Q88WN3	Ribosomal Protein L27	up	-	-	-
A0A165VYQ7	Ribosomal Protein S1	up	-		-
A0A3B6EL93	Ribosomal Protein L28	up	-	up	-
A0A3B6AUJ9	Ribosomal Protein S3	down	up	-	-
E2F3W4	Ribosomal Protein S15A	down	up	-	-
A0A3B6TBF6	Ribosomal Protein L9	down	up	down	down
A0A2X0S8T6	Ribosomal Protein L14	down	-	-	-
A0A3B5ZQF2	Ribosomal Protein L18	down	-	-	-
A0A3B6GLN8	Ribosomal Protein S5	down	up	-	-
A0A3B6SID4	Ribosomal Protein L7A	down	down	-	-
Q5I7K5	Ribosomal Protein LP1	down	up	down	-
Q5I7L3	Ribosomal Protein L10A	down	-	down	-
A0A1W5RML9	Ribosomal Protein SA	down	-	-	-
W5G990	Ribosomal Protein L31	down	up	-	-
P07280	Ribosomal Protein S19	down	up	down	up
W5EP45	Ribosomal Protein S25	down	-	-	-
W5E8X2	Ribosomal Protein S28	down	-	down	-
W5FEZ3	Ribosomal Protein S12	-	-	down	-
Q5I7K2	Ribosomal Protein S7	down	up	-	-
A0A3B6ISR9	Ribosomal Protein S21	down	down	-	down

## Data Availability

Genetic sequencing data have been deposited to the National Center of Biotechnology Information under SAR succession number SRP305571. The mass spectrometry proteomics data have been deposited to the ProteomeXchange Consortium via the IProX partner repository with the dataset identifier PXD024265.

## References

[1] Oshiro M., Zendo T., Nakayama J. (2021). Diversity and dynamics of sourdough lactic acid bacteriota created by a slow food fermentation system. J. Biosci. Bioeng..

[2] Lau S.W., Chong A.Q., Chin N.L., Talib R.A., Basha R.K. (2021). Sourdough microbiome comparison and benefits. Microorganisms.

[3] Menezes L.A.A., SavoSardaro M.L., Duarte R.T.D., Mazzon R.R., Neviani E., Gatti M., De Dea Lindner J. (2020). Sourdough bacterial dynamics revealed by metagenomic analysis in Brazil. Microorganisms.

[4] De Vuyst L., Neysens P. (2005). The sourdough microflora: Biodiversity and metabolic interactions. Trends Food Sci. Tech..

[5] Zhu J., Huang R., Hu D.X., Dou Y., Ren H.Y., Yang Z.X., Deng C., Xiong W., Wang D., Mao Y. (2019). Individualized prediction of metastatic involvement of lymph nodes posterior to the right recurrent laryngeal nerve in papillary thyroid carcinoma. OncoTargets Ther..

[6] Zhang G.H., Tu J., Sadiq F.A., Zhang W.Z., Wang W. (2019). Prevalence, genetic diversity, and technological functions of the *Lactobacillus sanfranciscensis* in sourdough: A Review. Compr. Rev. Food Sci. Food Saf..

[7] Chavan R.S., Chavan S.R. (2011). Sourdough technology-a traditional way for wholesome foods: A review. Compr. Rev. Food Sci. Food Saf..

[8] De Vuyst L., Harth H., Van Kerrebroeck S., Leroy F. (2016). Yeast diversity of sourdoughs and associated metabolic properties and functionalities. Int. J. Food Microbiol..

[9] Pontonio E., Di Cagno R., Mahony J., Lanera A., De Angelis M., van Sinderen D., Gobbetti M. (2017). Sourdough authentication: Quantitative PCR to detect the lactic acid bacterial microbiota in breads. Sci. Rep..

[10] Pulvirenti A., Solieri L., Gullo M., De Vero L., Giudici P. (2004). Occurrence and dominance of yeast species in sourdough. Lett. Appl. Microbiol..

[11] De Vuyst L., Van Kerrebroeck S., Harth H., Huys G., Daniel H.M., Weckx S. (2014). Microbial ecology of sourdough fermentations: Diverse or uniform?. Food Microbiol..

[12] Van Rossum H.M., Kozak B.U., Niemeijer M.S., Duine H.J., Luttik M.A.H., Boer V.M., Kotter P., Daran J.M.G., van Maris A.J.A., Pronk J.T. (2016). Alternative reactions at the interface of glycolysis and citric acid cycle in *Saccharomyces cerevisiae*. Fems. Yeast Res..

[13] Fujimoto A., Ito K., Narushima N., Miyamoto T. (2019). Identification of lactic acid bacteria and yeasts, and characterization of food components of sourdoughs used in Japanese bakeries. J. Biosci. Bioeng..

[14] Boyaci Gunduz C.P., Gaglio R., Franciosi E., Settanni L., Erten H. (2020). Molecular analysis of the dominant lactic acid bacteria of chickpea liquid starters and doughs and propagation of chickpea sourdoughs with selected Weissella confusa. Food Microbiol..

[15] Abedfar A., Hosseininezhad M., Corsetti A. (2019). Effect of wheat bran sourdough with exopolysaccharide producing *Lactobacillus plantarum* (NR_104573.1) on quality of pan bread during shelf life. LWT.

[16] Mihhalevski A., Sarand I., Viiard E., Salumets A., Paalme T. (2011). Growth characterization of individual rye sourdough bacteria by isothermal microcalorimetry. J. Appl. Microbiol..

[17] Teleky B.E., M artău A.G., Ranga F., Chețan F., Vodnar D.C. (2020). Exploitation of lactic acid bacteria and baker’s yeast as single or multiple starter cultures of wheat flour dough enriched with soy flour. Biomolecules.

[18] Thiele C., Gänzle M.G., Vogel R.F. (2002). Contribution of sourdough Lactobacilli, yeast, and cereal enzymes to the generation of amino acids in dough relevant for bread flavor. Cereal. Chem..

[19] Sieuwerts S., Bron P.A., Smid E.J. (2018). Mutually stimulating interactions between lactic acid bacteria and *Saccharomyces cerevisiae* in sourdough fermentation. LWT.

[20] Xu D., Zhang Y., Tang K.X., Hu Y., Xu X.M., Ganzle M.G. (2019). Effect of mixed cultures of yeast and Lactobacilli on the quality of wheat sourdough bread. Front. Microbiol..

[21] Dymond J.S. (2013). *Saccharomyces cerevisiae* growth media. Methods Enzymol..

[22] Hayek S.A., Gyawali R., Aljaloud S.O., Krastanov A., Ibrahim S.A. (2019). Cultivation media for lactic acid bacteria used in dairy products. J. Dairy Res..

[23] Mantzourani I., Plessas S., Odatzidou M., Alexopoulos A., Galanis A., Bezirtzoglou E., Bekatorou A. (2019). Effect of a novel *Lactobacillus paracasei* starter on sourdough bread quality. Food Chem..

[24] Ravi R.K., Walton K., Khosroheidari M. (2018). MiSeq: A next generation sequencing platform for genomic analysis. Methods Mol. Biol..

[25] Wang X., Li X., Deng X., Han H., Shi W., Li Y. (2007). A protein extraction method compatible with proteomic analysis for the euhalophyte Salicornia europaea. Electrophoresis.

[26] Cortés-Ríos J., Zárate A.M., Figueroa J.D., Medina J., Fuentes-Lemus E., Rodríguez-Fernández M., Aliaga M., López-Alarcón C. (2020). Protein quantification by bicinchoninic acid (BCA) assay follows complex kinetics and can be performed at short incubation times. Anal. Biochem..

[27] Vrancken G., De Vuyst L., Rimaux T., Allemeersch J., Weckx S. (2011). Adaptation of *Lactobacillus plantarum* IMDO 130201, a wheat sourdough isolate, to growth in wheat sourdough simulation medium at different pH values through differential gene expression. Appl. Environ. Microb..

[28] Teleky B.E., Martău G.A., Vodnar D.C. (2020). Physicochemical effects of *Lactobacillus plantarum* and *Lactobacillus casei* cocultures on soy–wheat flour dough fermentation. Foods.

[29] Penido F.C.L., de Oliveira Goulart C., Galvão Y.C.F., Teixeira C.V., de Oliveira R.B.P., Borelli B.M., Guimarães G.M., Neumann E., Sande D., de Araújo R.L.B. (2019). Antagonistic lactic acid bacteria in association with *Saccharomyces cerevisiae* as starter cultures for standardization of sour cassava starch production. J. Food Sci. Technol..

[30] García Vilanova M., Díez C., Quirino B., Iñaki Álava J. (2015). Microbiota distribution in sourdough: Influence of High sucrose resistant strains. Int. J. Gastron. Food Sci..

[31] Richardson G., Langton M., Bark A., Hermansson A.M. (2003). Wheat starch gelatinization—the effects of sucrose, emulsifier and the physical state of the emulsifier. Starke.

[32] Kang M.J., Bae I.Y., Lee H.G. (2018). Rice noodle enriched with okara: Cooking property, texture, and in vitro starch digestibility. Food Biosci..

[33] Meng J., Zhao W. (2005). Properties and applications of trehalose in new food development. Food Sci..

[34] Zhang X.R., Zhang Y.X., Li H. (2020). Regulation of trehalose, a typical stress protectant, on central metabolisms, cell growth and division of *Saccharomyces cerevisiae* CEN.PK113-7D. Food Microbiol..

[35] Nam Y.W., Nishimoto M., Arakawa T., Kitaoka M., Pushinobu S. (2019). Structural basis for broad substrate specificity of UDP-glucose 4-epimerase in the human milk oligosaccharide catabolic pathway of *Bifidobacterium longum*. Sci. Rep..

[36] Li C., Cai W.T., Liu S.L., Zhou C.H., Cao M.Y., Yin H.W., Sun D.X., Zhang S.L., Loor J.J. (2020). Association of UDP-galactose-4-epimerase with milk protein concentration in the Chinese Holstein population. Asian-Australas. J. Anim. Sci..

[37] Zhang S.S., Xu Z.S., Qin L.H., Kong J. (2020). Low-sugar yogurt making by the co-cultivation of *Lactobacillus plantarum* WCFS1 with yogurt starter cultures. J. Dairy Sci..

[38] Heinisch J.J., Boles E., Timpel C. (1996). A yeast phosphofructokinase insensitive to the allosteric activator fructose 2,6-bisphosphate -Glycolysis metabolic regulation allosteric control. J. Biol. Chem..

[39] Hussain S., Ali S., Mumtaz S., Shakir H.A., Ahmad F., Tahir H.M., Ulhaq M., Khan M.A., Zahid M.T. (2020). Dose and duration-dependent toxicological evaluation of lead acetate in chicks. Environ. Sci. Pollut. R..

[40] Heitmann M., Zannini E., Arendt E. (2018). Impact of *Saccharomyces cerevisiae* metabolites produced during fermentation on bread quality parameters: A review. Crit. Rev. Food Sci. Nutr..

[41] Corsetti A., Settanni L. (2007). Lactobacilli in sourdough fermentation. Food Res. Int..

[42] Salim-ur-Rehman, Paterson A., Piggott J.R. (2006). Flavour in sourdough breads: A review. Trends Food Sci. Tech..

[43] Su X.Q., Wu F.F., Zhang Y.Q., Yang N., Chen F., Jina Z.Y., Xu X.M. (2019). Effect of organic acids on bread quality improvement. Food Chem..

[44] Kieliszek M., Pobiega K., Piwowarek K., Kot A.M. (2021). Characteristics of the proteolytic enzymes produced by lactic acid bacteria. Molecules.

[45] Waśko A., Kieliszek M., Targoński Z. (2012). Purification and characterization of a proteinase from the probiotic *Lactobacillus rhamnosus* OXY. Prep. Biochem. Biotechnol..

[46] Vermeulen N., Ganzle M.G., Vogel R.F. (2006). Influence of peptide supply and cosubstrates on phenylalanine metabolism of *Lactobacillus sanfranciscensis* DSM20451(T) and *Lactobacillus plantarum* TMW1.468. J. Agric. Food Chem..

[47] Fernandez M., Zuniga M. (2006). Amino acid catabolic pathways of lactic acid bacteria. Crit. Rev. Microbiol..

[48] Fernandez M., Rodriguez A., Fulco M., Soteras T., Mozgovoj M., Cap M. (2020). Effects of lactic, malic and fumaric acids on Salmonella spp. counts and on chicken meat quality and sensory characteristics. J. Food Sci. Technol..

[49] Mocibob M., Weygand-Durasevic I. (2008). The proximal region of a noncatalytic eukaryotic seryl-tRNA synthetase extension is required for protein stability in vitro and in vivo. Arch. Biochem. Biophys..

[50] Majid M., Khan M.S., Mustafa G., Salam A. (2019). Structural and functional analysis of RPL16 a large ribosomal subunit protein of plastid translational machinery in plants. Int. J. Agric. Biol..

[51] Ting Y.H., Lu T.J., Johnson A.W., Shie J.T., Chen B.R., Suresh S.S., Lo K.Y. (2017). Bcp1 is the nuclear chaperone of Rpl23 in *Saccharomyces cerevisiae*. J. Biol. Chem..

[52] Ciplys E., Zitkus E., Gold L.I., Daubriac J., Pavlides S.C., Hojrup P., Houen G., Wang W.A., Michalak M., Slibinskas R. (2015). High-level secretion of native recombinant human calreticulin in yeast. Microb. Cell Factories.

